# Concomitant Parenchymal, Subarachnoid, and Ventricular Neurocysticercosis in Rome, Italy: A Case Report with a 4-Year Follow-Up

**DOI:** 10.3390/tropicalmed9080187

**Published:** 2024-08-21

**Authors:** Maria Letizia Giancola, Shalom Haggiag, Angela Corpolongo, Alessandro Stasolla, Andrea Mariano, Agazio Menniti, Paolo Campioni, Barbara Bartolini, Pierluigi Galizia, Antonella Vulcano, Carla Fontana, Claudio Gasperini, Elise O’Connell, Hector H. Garcia, Theodore E. Nash, Emanuele Nicastri

**Affiliations:** 1 Clinical Department, National Institute for Infectious Diseases “Lazzaro Spallanzani”, Istituto di Ricovero e Cura a Carattere Scientifico (IRCCS), 00149 Rome, Italy; mletizia.giancola@inmi.it (M.L.G.); andrea.mariano@inmi.it (A.M.); emanuele.nicastri@inmi.it (E.N.); 2 Department of Neuroscience, Azienda Ospedaliera San Camillo Forlanini, 00152 Rome, Italy; neuroshalom@hotmail.com (S.H.); pl.galizia@gmail.com (P.G.); c.gasperini@libero.it (C.G.); 3 Neuroradiology Unit, Azienda Ospedaliera San Camillo Forlanini, 00152 Rome, Italy; astasolla@scamilloforlanini.rm.it; 4 Neurosurgery Unit, Azienda Ospedaliera San Camillo Forlanini, 00152 Rome, Italy; letiz1@yahoo.it; 5 Radiology Unit, National Institute for Infectious Diseases “Lazzaro Spallanzani”, Istituto di Ricovero e Cura a Carattere Scientifico (IRCCS), 00149 Rome, Italy; paolo.campioni@inmi.it; 6 Laboratory of Microbiology and Biological Bank, National Institute of Infectious Diseases “L. Spallanzani”, Istituto di Ricovero e Cura a Carattere Scientifico (IRCCS), 00149 Rome, Italy; barbara.bartolini@inmi.it (B.B.); antonella.vulcano@inmi.it (A.V.); carla.fontana@inmi.it (C.F.); 7 Laboratory of Parasitic Diseases, National Institute of Allergy and Infectious Diseases, National Institutes of Health, 4 Center Drive, Building 4, Bethesda, MD 20892, USAtnash@niaid.nih.gov (T.E.N.); 8 Center for Global Health, Universidad Peruana Cayetano Heredia, and Cysticercosis Unit, Instituto Nacional de Ciencias Neurologicas, Av. Honorio Delgado 430, San Martin de Porres, Lima 15102, Peru; hgarcia1@jhu.edu

**Keywords:** *Taenia solium*, parenchymal neurocysticercosis, subarachnoid neurocysticercosis, ventricular neurocysticercosis, brain infection, quantitative polymerase chain reaction, Next Generation Sequencing, neglected tropical disease

## Abstract

Neurocysticercosis (NCC) is caused by the larval stage of *Taenia solium*. This parasitic disease is endemic in many areas of the world and is emerging in Europe. NCC can affect different brain regions, but simultaneous involvement of the parenchymal, subarachnoid, and ventricular regions is rare. We report the case of a 39-year-old woman from Honduras, resident in Rome for 10 years, who presented to the Emergency Department complaining of headaches, transient hemianopsia, and bilateral papilledema. MRI showed a concomitant parenchymal, subarachnoid, and ventricular involvement in the brain. *T. solium* IgG antibodies were detected in the blood. The etiological diagnosis of NCC was obtained by identifying *T. solium* in cerebrospinal fluid using Next Generation Sequencing. Endoscopic neurosurgery with the placement of a ventricular shunt and medical long-term anti-parasitic treatment with a cumulative number of 463 days of albendazole and 80 days of praziquantel were performed. A successful 4-year follow-up is reported. NCC is one of the most common parasitic infections of the human CNS, but it is still a neglected tropical disease and is considered to be an emerging disease in Europe. Its diagnosis and clinical management remain a challenge, especially for European clinicians.

## 1. Introduction

Neurocysticercosis (NCC) is caused by the larval stage (cysticercus) of the pork tapeworm *Taenia solium* (*T. solium*). This parasitic disease affects the central nervous system (CNS) and is endemic in many areas of the world, including Asia, sub-Saharan Africa, and Latin American countries.

According to the Pan American Health Organisation, NCC affects 14.9 million people in the Americas [1], making it one of the most frequent helminthic infections of the CNS. In endemic areas, seroprevalence for anti-*T. solium* antibodies ranges from 2 to 30% [2]. In these regions, *T. solium* is also the main cause of acquired epilepsy, contributing to approximately one-third of cases, ranging from 0.5 up to 67.6% in a published systematic review and meta-analysis [3]. In the U.S., around 2000 patients are hospitalized for NCC each year, and around 5000 cases are diagnosed [4]. Due to the increase in migration and travel, *Taenia* cases have emerged in Europe, with estimated prevalence ranging from 0.02 to 0.67%, with the lowest reported in Italy (0.02–0.04%), and only one human cysticercosis case was suspected as an autochthonous among those published [5]. Cases in European countries with low NCC prevalence are often found in travelers or migrants from endemic regions [6].

NCC can affect different brain regions, and the clinical symptoms can vary depending on the number of cysts present, their location, and the resulting inflammation. Simultaneous involvement of the parenchymal, subarachnoid, and ventricular regions is rare, particularly in Europe, and can be linked to a severe prognosis [6].

This case involves a 39-year-old woman from Honduras who resides in Rome. She was diagnosed with NCC affecting the parenchymal, subarachnoid, and ventricular regions.

## 2. Case Presentation

A 39-year-old woman from Honduras, residing in Rome for 10 years, presented to the Emergency Department on 16 May 2020 with complaints of headaches, transient hemianopsia, and bilateral papilledema. A brain CT scan revealed minute meningeal calcifications in the right parietal region, parenchymal lesions, and tetraventricular hydrocephalus.

**Figure 1:**
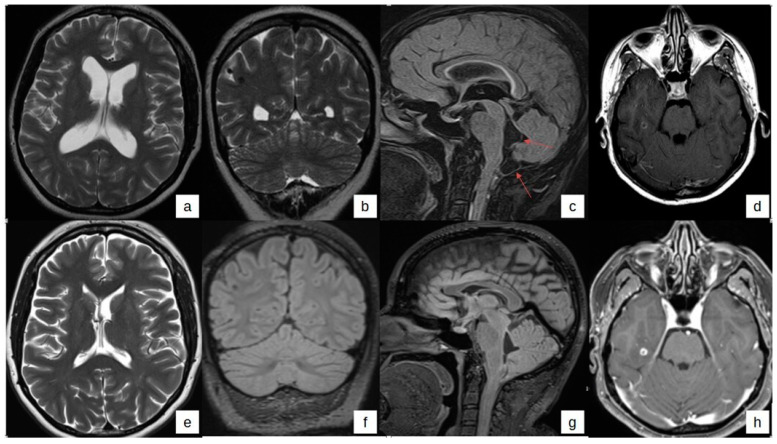
Brain MR imaging at diagnosis (**a**–**d**) and follow-up (**e**–**h**). (**a**–**d**). MR images obtained in 2020 using T2-weighted axial, T2-weighted coronal, T1-FLAIR sagittal with contrast, and T1-weighted with contrast axial sequences. Findings include dilatation of the supratentorial ventricular system (**a**), subcortical localization in the right parietal region (**b**), pachymeningeal enhancement observed in the dilated cisterna magna and cervical spine (**c**) (the red arrows indicate cysts in the fourth ventricle and cisterna magna), and a small subcortical localization with ring enhancement in the right temporal region (**d**–**h**). MR images obtained in 2023 using T2-weighted axial, T2-weighted coronal, T1-FLAIR sagittal with contrast, and T1-weighted with contrast axial sequences. Notable changes include regression of ventricular dilatation (**e**), persistent subcortical localization in the right parietal region (**b**), collapse of the cisterna magna with reduced pachymeningeal enhancement (**g**), and unchanged subcortical localization in the right temporal region with ring enhancement (**h**).

The patient had a history of epilepsy since the age of 17, for which she was on anti-epileptic therapy for approximately 4 years, and she also experienced occasional headaches. She was then transferred to the Neurology department, where a brain MRI confirmed the presence of parenchymal lesions (one of them 6 mm in size in the right temporal region with contrast enhancement and two calcific lesions) and a cyst in the fourth ventricle that expanded out downwards to the cisterna magna and tetraventricular hydrocephalus (Figure 1a–d). A spine MRI showed a faint enhancement in the perimedullary region extending from the craniocervical junction to the fifth cervical vertebra. Chest and abdomen CT scans were normal.

Blood tests detected *T. solium* IgG antibodies (ELISA TAENIA SOLIUM IgG, NovaLisa, NovaTec Immunodiagnostica GmbH, Germany, and Western blot IgG CYSTICERCOSIS, LDBIO Diagnostics, Lyon, France), while tests for Quantiferon TB Gold, Widal and Wright reactions, HIV, *Trypanosoma cruzi*, and *Treponema pallidum* were negative. No parasites were found in the stool samples. On 19 May 2020, an analysis of the cerebrospinal fluid (CSF) showed low glucose level (12 mg/dL), high protein levels (110 mg/dL), and an increased number of cells (105/mm^3^) with histiocytes, some neutrophils, and few lymphocytes; there were no eosinophils (Table 1). A home-made polymerase chain reaction (PCR) test for *T. solium* was negative. For this purpose, 1 mL of CSF was pelleted, and the pellet was extracted with QIAamp DNA Mini Kits (Qiagen, Hilden, Germany) and eluted in 50 μL of elution buffer. A conventional end-point PCR was performed using, as target region, a mitochondrial sequence of a 314 bp fragment of the mitochondrial 12S rRNA gene [7]. PCRs for *M. tuberculosis*, *Toxoplasma gondii*, *Listeria monocytogenes*, *Treponema pallidum*, and *Aspergillus* on CSF were also negative. No bacteria were detected using microscopic examination, and the Filmarray PCR assay for neutotropic pathogens (including various bacterial, viral, and fungal agents), Cryptococcus neoformans antigens, and HIV-RNA on CSF were all negative. The patient was started on a four-drug regimen for tuberculosis along with steroids, but it was discontinued 21 days later when Next Generation Sequencing (NGS) identified *T. solium* in the CSF (Table 1). For NGS, 140 µL of CSF were extracted using a QIAmp viral RNA minikit (Qiagen) following the manufacturer’s instructions and were eluted in 30 μL of water. Reverse transcription and Sequence Independent Single-Primer Amplification (SISPA) were performed [8]. The obtained DNA was purified and the shotgun sequencing run was performed by using the Ion Torrent S5 platform (Thermofisher). On 18 June 2020, *T. solium* IgG (Western blot IgG CYSTICERCOSIS, LDBIO Diagnostics, Lyon, France) resulted in a positive result for CSF.

On 6 July 2020, an endoscopic neurosurgery with placement of a ventricular shunt was performed, but the removal of the fourth ventricle cyst was not possible because of the strong attachment to the wall due to severe inflammation. On 8 July 2020, the patient initiated oral albendazole treatment (400 mg twice daily, taken for a cumulative total of 463 days), along with a one-month course of steroid therapy (desamethasone 0.1 mg/kg four times daily, tapered gradually). Additionally, considering the patient’s prior history of epilepsy, the diagnosis of NCC, and the ventricular shunt procedure (later ventriculoperitoneal shunt), anticonvulsant medication (levetiracetam 500 mg twice daily) was added, despite the absence of recent seizures.

MRI scans performed in February and April 2021 documented an increased number of lesions. Upon a more thorough retrospective analysis, these lesions were found to have been already present in the earlier images from February and April 2020, revealing a greater number of hyperintensity lesions within the subarachnoid spaces and cysts, including the cisterna magna (Figure 1). Moreover, on 26 April 2021, a further increase in CSF protein levels (1112 mg/dL), along with very high levels of *T. solium* antigen in the CSF and negative *T. solium* antigen in serum (Institute of Tropical Medicine, Antwerp), were found. On April 2021, levetiracetam was discontinued and replaced with zonisamide because of poor tolerability. An international panel of experts on NCC was consulted; albendazole therapy was continued, and praziquantel (60 mg/Kg/day, 3000 mg/day) was added from 19 May 2021, until 7 August 2021, when the patient stopped it on her own due to abdominal pain. Prednisone at the dose of 50 mg/day was started on 5 May 2021 and then gradually reduced. Between September and October 2021, improvements were observed in MRI scans, with a decrease in subarachnoid signal intensity and improvement in CSF parameters. The *T. solium* antigen in CSF measured 14.5 ng/mL, and a quantitative polymerase chain reaction (qPCR, Tsol1R13 qPCR) for *T. solium* was negative in both CSF and plasma (NIH Laboratories Bethesda, Bethesda, MD, USA) (Table 1) [9]. Albendazole was stopped on 14 November 2021 and prednisone was tapered off a few days earlier. After that, the patient went back to her job as a babysitter and returned to her home country for about a year. Currently, she is in good clinical condition and has no symptoms, and she has annual follow-up visits. The last follow-up assessment showed a slight reduction in CSF protein levels on 22 August 2023 and stable brain MRI scans in September 2023. The qPCR for *T. solium* on CSF and plasma were undetectable, and the concentration of *Taenia* antigen remained undetectable in CSF and blood (Table 1). At the NIH Laboratory, the initial CSF samples (collected on 18 June 2020) were retrospectively tested, and qPCR was found to be positive (value 24.6 quantification cycle, Cq), with a CSF *Taenia* antigen concentration of 99,800 ng/mL (negative reference range 0- < 1 ng/mL). The patient remains under observation.

Table 1 summarizes the changes in CSF and blood parameters and the prescribed treatment. Figure 1 displays brain MR images at diagnosis (a–d) and during follow-up (e–h).

## 3. Discussion

We report a case of NCC with combined involvement of the parenchymal, ventricles, and subarachnoid spaces of the brain, as well as the spinal cord. Involvement of all three brain compartments is rare in Italy and required long-term treatment. The patient was successfully treated for a cumulative number of 463 days with albendazole and 80 days with praziquantel. It is worth noting that she still had a viable ventricular NCC 20 years after symptoms’ onset and 10 years from the date of immigration.

NCC is a Neglected Tropical Disease (NTD) endemic in certain parts of the world, but relatively rare in Europe. The most common form of NCC is its parenchymal form, whereas its ventricular and subarachnoid involvement is less frequent. In large series of cases followed for several years in the USA, ventricular cysts were found in 19% of 121 cases and the subarachnoid form in 25.4% of 134 cases [10,11]. Nevertheless, changes in current epidemiological trends in NCC indicate that extra-parenchymal NCC is becoming more prevalent [12]. Furthermore, some evidence suggests the possibility of a more recent infection of patients affected by parenchymal and ventricular NCC than subarachnoid NCC, probably related to a longer preclinical phase of extra-parenchymal NCC, explaining the older age of patients with subarachnoid NCC [13].

The simultaneous involvement of parenchymal, subarachnoid, and ventricular regions is uncommon. Only 2 out of 46 (4.3%) imported cysticercosis cases in Spain had multiple types of NCC in a single patient [14]. This condition can indicate a poor prognosis and significant difficulties for diagnosis and treatment.

Cysts located in different compartments of the brain cause different clinical manifestations and need different clinical approaches, either medical or surgical. The infection of the parenchymal tissue often causes convulsions. Subarachnoid cysts cause symptoms related to mass effect, chronic arachnoiditis resulting in infarction and hydrocephalus, while ventricular cysts primarily cause symptoms due to the obstruction of CSF flow and associated ventriculitis.

Despite presenting with all three localizations of NCC simultaneously, our patient complained of only headache and a transient visual impairment, with a history of epilepsy in the past.

Diagnosing NCC was challenging in our case, as the patient’s origin and anti-*T solium* antibodies were suggestive, but not enough to confirm it. Several studies have reported a high sensitivity of *T. solium* PCR in CSF, but not 100% [15]. The home-made PCR test performed at our Institute was negative because of its low sensitivity, and more innovative and advanced methods like NGS were employed for diagnosis [8,16]. The qPCR on the CSF collected at diagnosis and retrospectively tested at NIH was positive, probably because of different sensitivity between the two target regions used: a mitochondrial sequence (314 bp fragment of the mitochondrial 12S rRNA gene) at INMI, and a very small and highly repetitive interspersed (non-coding) region, which is probably more sensitive, at NIH [9].

Assays for the detection of parasite antigen in CSF, serum, and even urine, may also be useful to confirm the diagnosis, but they are not currently widely available commercially and are less sensitive than assays for specific IgG. Positive results correlate with the number of viable cysticerci [17].

In addition, the diagnostic process faced problems because of its time-consuming and expensive nature and the limited number of international reference laboratories with sensitive and specific techniques for direct diagnosis of NCC. In fact, antigen and highly sensitive PCR are performed only in a few reference laboratories.

We can argue that NGS was probably not essential for the diagnosis of NCC in this case; however, it was a very important support for us, as antibody detection in blood was insufficient for diagnosis. In fact, serology positivity in blood simply reflects exposure to parasites and cannot be used as the only diagnostic criterion. Furthermore, detection of specific IgG in CSF could results from passive diffusion from blood. Indeed, the diagnosis of NCC is often made using serologic tests combined with imaging features. However, imaging is often nonspecific and abnormalities can be missed by standard sequences of MRI [18], as in our patient, in a non-endemic country, where experience with this disease is limited. Therefore, NGS was a great support for diagnosis and could be performed within our laboratory, while qPCR and antigen determination of *T. solium* were only available in reference laboratories abroad.

We found some difficulties in identifying lesions located in the subarachnoid space and basal cistern using the classical sequences (Fluid Attenuation Inversion Recovery (FLAIR) and T1). In fact, only a retrospective analysis of the imaging of our case showed the early involvement of these compartments. The usefulness is known of 3D MRI with FIESTA sequences compared to classical sequences to diagnose neurocysticercosis located in the basal cisterns of the subarachnoid space in patients with a suspected diagnosis of extra parenchymal NCC. These sequences should be routinely used to favor an earlier diagnosis and improve the prognosis of patients affected by this severe form of the disease [18].

Moreover, low CSF glucose levels at diagnosis led to the initial consideration, and subsequent exclusion, of tuberculosis. In tropical settings, NCC and tuberculosis need to be considered in all differential diagnoses of neurological syndromes with very low glucose levels in CSF and brain masses [19].

The clinical outcome of NCC differs according to its location: ventricular and subarachnoid involvement more often leads to neurological complications, long-term consequences, relevant morbidity, and mortality. Subarachnoid NCC often causes severe sequelae: in a large series of patients diagnosed and followed up at the NIH, only 42.4% of the patients were in good health conditions without problems, as in our patient, and 24.2% of them were unable to work [11]. In intraventricular NCC, 15% had severe disability, as reported in one of the largest reported series [10].

The therapeutic management of the patient presented considerable challenges. Guidelines for the clinical management of NCC are available [20], but the strength of recommendations and the quality of evidence are sometimes weak. The treatment for parenchymal disease is fairly standard; ventricular disease requires removing the cyst or bypassing the obstruction, and the treatment of ventriculitis is sometimes necessary. Although the treatment of ventricular disease has not been subjected to randomized studies, there seems to be general agreement on what is effective treatment. In our patient, as in others, the management of subarachnoid disease posed the greatest difficulties. There is no randomized clinical trial and consensus on how to treat subarachnoid disease, which drugs to use, if mono- or combined therapy should be used, and for how long. However, recent studies indicate that treatment with cysticidal agents can be effective if used for a long time and with careful testing of taenia markers (taenia antigen and/or *T. solium* DNA) in CSF, and sometimes in plasma, which can help to define the duration of treatment to prevent parasite regrowth [9]. The subarachnoid disease needs long-term treatment because of both the ability of this form to grow and the risk of clinical relapse even decades after insufficient treatment. Expert opinions suggest that the type and duration of antiparasitic treatment should be decided individually by the absence of parasite antigens or DNA in CSF.

In fact, antigens in CSF and plasma and DNA in CSF are considered to be a marker of disease activity along with the normalization of cellularity and hypoglycorrachia in CSF, and should be monitored in the follow up. They are a useful tool for the decision to discontinue cysticidal treatment and steroids in subarachnoid NCC [9,20].

In the successful management of this case, the international experts’ scientific board played a pivotal role. Effective management required a comprehensive multidisciplinary approach, involving specialists in infectious diseases, neurology, neurosurgery, neuroradiology, microbiology, and a multimodal strategy. This included intensified and combined antiparasitic therapy with higher and longer doses than standard protocols, together with anti-inflammatory interventions.

Drug availability posed an additional challenge in NCC treatment, with praziquantel not being registered in Italy and albendazole, although registered, presenting availability challenges for a long off-label duration.

We will continue to closely monitor the disease activity with ongoing long-term follow-up visits of the patient.

## 4. Conclusions

NCC is one of the most prevalent parasitic infections of human CNS, but it is still a NTD, according to the WHO in 2016, and it is considered to be an emerging disease in Europe. Its clinical management remains a challenge for European clinicians.

## Figures and Tables

**Table 1:** Progression of CSF parameters of the patient at diagnosis and follow-up, along with the corresponding therapeutic interventions administered.

	19 May 2020	1 June 2020	18 June 2020	26 April 2021	28 June 2021	15 October 2021	17 June 2022	22 August 2023
WBC (n/mm^3^)	105 (N 40%, L 10%, histiocytes)	80 (N 40%, L 40%)	20 (L 70%)	20 (L 40%, histiocytes 40%)	11	14 (L 70%, N 10%)	8	7 (L 70%)
Glucose (mg/dL)	12	5	23	54	59	48	56	54
Proteins (mg/dL)	110	120	88	1112	736	837	808	658
Home made PCR for *T. solium* (INMI)	negative			negative		negative	negative	
NGS (INMI)			positive for *T. solium*					
*T. solium* Ag concentration (ng/mL)			* 99800 (NIH)	highly positive (ITG)	highly positive (ITG)	14.5 (NIH)		negative (NIH)
qPCR for *T. solium* (NIH)			* positive (value 24.6 Cq)			negative		negative
Therapy							-	-
- Anti-TB	yes	yes	no	no	no	no		
- Steroids	no	yes	yes	no	no	yes		
- Albendazole	no	no	yes	yes	yes	yes		
- Praziquantel	no	no	no	no	yes	no		

Abbreviations: WBC = white blood cells; CSF = cerebrospinal fluid; N = neutrophils; L = lymphocytes; E = eosinophils; NGS = Next Generation Sequencing; *T. solium* = *Taenia solium*; PCR = polymerase chain reaction; Ag = antigen; TB = tuberculosis; INMI = National Institute for Infectious Diseases “L. Spallanzani”, Rome, Italy; ITG = Institute of Tropical Medicine, Antwerp, Belgium; NIH = National Institutes of Health, Bethesda, USA. Cq = Quantification Cycle * Retrospectively tested.

## Data Availability

The datasets used during the current study are available from the corresponding author on reasonable request.

## References

[1] https://www.paho.org/en/topics/taenia-solium-taeniasiscysticercosis.

[2] Ndimubanzi P.C., Carabin H., Budke C.M., Nguyen H., Qian Y.J., Rainwater E., Dickey M., Reynolds S., Stoner J.A. (2010). A systematic review of the frequency of neurocyticercosis with a focus on people with epilepsy. PLoS Negl. Trop. Dis..

[3] Debacq G., Moyano L.M., Garcia H.H., Boumediene F., Marin B., Ngoungou E.B., Preux P.-M. (2017). Systematic review and meta-analysis estimating association of cysticercosis and neurocysticercosis with epilepsy. PLoS Negl. Trop. Dis..

[4] O’Neal S.E., Flecker R.H. (2015). Hospitalization frequency and charges for neurocysticercosis, United States, 2003–2012. Emerg. Infect. Dis..

[5] Laranjo-González M., Devleesschauwer B., Trevisan C., Allepuz A., Sotiraki S., Abraham A., Afonso M.B., Blocher J., Cardoso L., da Costa J.M.C. (2017). Epidemiology of taeniosis/cysticercosis in Europe, a systematic review: Western Europe. Parasit. Vectors.

[6] Stelzle D., Abraham A., Kaminski M., Schmidt V., De Meijere R., Bustos J.A., Garcia H.H., Sahu P.S., Bobic B., Cretu C. (2023). Clinical characteristics and characteristics and management of neurocysticercosis patients: A retrospective assessment of case reports from Europe. J. Travel Med..

[7] von Nickisch-Rosenegk M., Lucius R., Loos-Frank B. (1999). Contributions to the phylogeny of the Cyclophyllidea (Cestoda) inferred from mitochondrial 12S rDNA. J. Mol. Evol..

[8] Djikeng A., Halpin R., Kuzmickas R., Depasse J., Feldblyum J., Sengamalay N., Afonso C., Zhang X., Anderson N.G., Ghedin E. (2008). Viral genome sequencing by random priming methods. BMC Genom..

[9] O’Connell E.M., Harrison S., Dahlstrom E., Nash T., Nutman T.B. (2020). A Novel, Highly Sensitive Quantitative Polymerase Chain Reaction Assay for the Diagnosis of Subarachnoid and Ventricular Neurocysticercosis and for Assessing Responses to Treatment. Clin. Infect. Dis..

[10] Nash T.E., Ware J.M., Mahanty S. (2018). Intraventricular Neurocysticercosis: Experience and Long-Term Outcome from a Tertiary Referral Center in the United States. Am. J. Trop. Med. Hyg..

[11] Nash T.E., O’Connell E.M., Hammoud D.A., Wetzler L., Ware J.M., Mahanty S. (2020). Natural History of Treated Subarachnoid Neurocysticercosis. Am. J. Trop. Med. Hyg..

[12] Hamamoto Filho P.T., Norcia L.F., Fleury A., Zanini M.A. (2024). Current Role of Surgery in the Treatment of Neurocysticercosis. Pathogens.

[13] Tellez-Arellano C.A., Kuschick-Fehér J., Romero-Gonzalez F.G., Fleury A. (2024). Neurocysticercosis: The Duration of Its Preclinical Phase Relies on the Parasite Location. Trop. Med. Int. Health.

[14] Herrador Z., Pérez-Molina J.A., Henríquez Camacho C.A., Rodriguez-Guardado A., Bosch-Nicolau P., Calabuig E., Domínguez-Castellano A., Pérez-Jacoiste M.A., de Guevara M.C.L., Mena A. (2020). REDIVI Study Group. Imported cysticercosis in Spain: A retrospective case series from the +REDIVI Collaborative Network. Travel Med. Infect. Dis..

[15] Yera H., Dupont D., Houze S., Ben M’rad M., Pilleux F., Sulahian A., Gatey C., Gay Andrieu F., Dupouy-Camet J. (2011). Confirmation and follow-up of neurocysticercosis by real-time PCR in cerebrospinal fluid samples of patients living in France. J. Clin. Microbiol..

[16] Fei X., Li C., Zhang Y., Zhang H., Liu X., Ji X., Shi Y., Liu N., Wu M., Du F. (2020). Clin Next-generation sequencing of cerebrospinal fluid for the diagnosis of neurocysticercosis. Neurol. Neurosurg..

[17] Fleury A., Garcia E., Hernández M., Carrillo R., Govezensky T., Fragoso G., Sciutto E., Harrison L.J., Parkhouse R.M. (2013). Neurocysticercosis: HP10 antigen detection is useful for the follow-up of the severe patients. PLoS Negl. Trop. Dis..

[18] Carrillo Mezo R., Lara García J., Arroyo M., Fleury A. (2015). Relevance of 3D Magnetic Resonance Imaging Sequences in Diagnosing Basal Subarachnoid Neurocysticercosis. Acta. Trop..

[19] Gothi R. (2013). Consider tuberculoma and cysticercosis in the differential diagnosis of brain tumour in tropical countries. BMJ.

[20] White A.C., Coyle C.M., Rajshekhar V., Singh G., Hauser W.A., Mohanty A., Garcia H.H., Nash T.E. (2018). Diagnosis and Treatment of Neurocysticercosis: 2017 Clinical Practice Guidelines by the Infectious Diseases Society of America (IDSA) and the American Society of Tropical Medicine and Hygiene (ASTMH). Clin. Infect. Dis..

